# Endoscopic spinal cord untethering using a 1 cm skin incision technique in pediatrics: a technical case report

**DOI:** 10.1186/s12887-023-04390-7

**Published:** 2023-11-29

**Authors:** Eitaro Ishisaka, Shigeyuki Tahara, Atsushi Tsukiyama, Toshiki Nozaki, Yujiro Hattori, Akio Morita, Yasuo Murai

**Affiliations:** 1 Department of Neurological Surgery, Nippon Medical School Musashi Kosugi Hospital, 1-383 Kosugimachi, Nakahara-ku, Kawasaki City, 211-8533 Kanagawa Japan; 2 Department of Neurological Surgery, Graduate School of Medicine, Nippon Medical School, Tokyo, Japan; 3 Department of Anatomy and Neurobiology, Graduate School of Medicine, Nippon Medical School, Tokyo, Japan; 4 Department of Neurological Surgery, Nippon Medical School, Bunkyo-ku, Tokyo, Japan

**Keywords:** Filum Terminale, Spinal lipoma, Tethered cord syndrome, Endoscopic untethering

## Abstract

**Background:**

Spinal cord untethering by sectioning the filum terminale is commonly performed in tethered cord syndrome patients with minor abnormalities such as filar lipoma, thickened filum terminale, and low conus medullaris. Our endoscopic surgical technique, using the interlaminar approach, allows for sectioning the filum terminale through a very small skin incision. To our knowledge, this procedure has not been previously reported. This is the first case report involving a 1 cm skin incision.

**Case presentation:**

A 9-month-old male patient was referred to our neurosurgical department due to a coccygeal dimple. MRI revealed a thickened fatty filum. After considering the treatment options for this patient, the parents agreed to spinal cord untethering. A midline 1 cm skin incision was made at the L4/5 vertebral level. Untethering by sectioning the filum terminale was performed by full endoscopic surgery using the interlaminar approach. The procedure was uneventful and there were no postoperative complications.

**Conclusions:**

In terms of visibility and minimizing invasiveness, our surgical technique of using the interlaminar approach with endoscopy allows for untethering by sectioning the filum terminale through a very small skin incision.

## Background

Filar lipoma, also known as fatty filum terminale or lipoma of the filum terminale, can cause tethered cord syndrome. Tethered cord syndrome can include symptoms such as sensory or motor deficits, urinary incontinence or urgency, back and leg pain, and scoliosis [1–3]. However, many cases are asymptomatic and are discovered because there are lumbar skin abnormalities [1, 4].

Surgical untethering is commonly performed in patients with symptomatic filar lipoma. However, for asymptomatic patients with minor abnormalities on magnetic resonance imaging (MRI), such as thickened filum terminale, filar lipoma, or low conus medullaris, there is still no consensus regarding surgical indications.

Since untethering for symptomatic filar lipomas has a 50–80% chance of improving symptoms, and some patients have sequelae of symptoms, prophylactic surgery in asymptomatic patients may be an option [3–7].

The classical surgical treatment of filar lipoma is one-level laminectomy or laminectomy and microsurgical sectioning of the filum terminale [8, 9]. To keep surgical invasiveness and complications to a minimum, minimally invasive techniques such as the interlaminar approach or endoscopic-assisted techniques have been increasingly used in recent years [5, 9–12]. We present our surgical technique for a minimally invasive full-endoscopic untethering that can be performed through a 1 cm skin incision, the smallest skin incision that has been reported to date.

## Case presentation

**Fig. 1:**
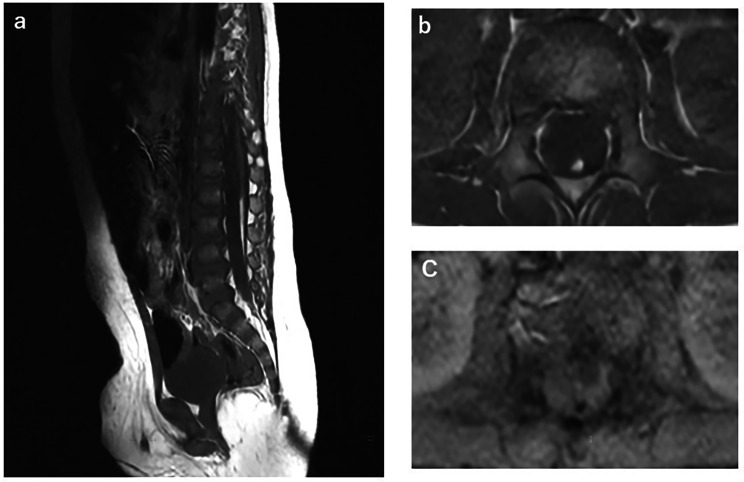
Preoperative MRI showed a T1 hyperintense fatty filum. **a**: sagittal T1-weighted image. **b**: axial T1-weighted image. **c**: signal suppression on fat T1 saturated sequence

A 9-month-old male patient was referred to our neurosurgical department due to a coccygeal dimple. The sacral hollow was 3.5 cm from the anus, above the gluteal cleft, with no redness or drainage. The patient had no neurological deficits. MRI was performed to check for possible spinal cord tethering and revealed a thickened fatty filum with a 3 mm diameter area of T1 hyperintensity (Fig. 1). Voiding cystourethrography showed no bladder morphology abnormalities or vesicoureteral reflux. After considering the treatment options for this patient, the parents agreed to spinal cord untethering.

### Operation

**Fig. 2:**
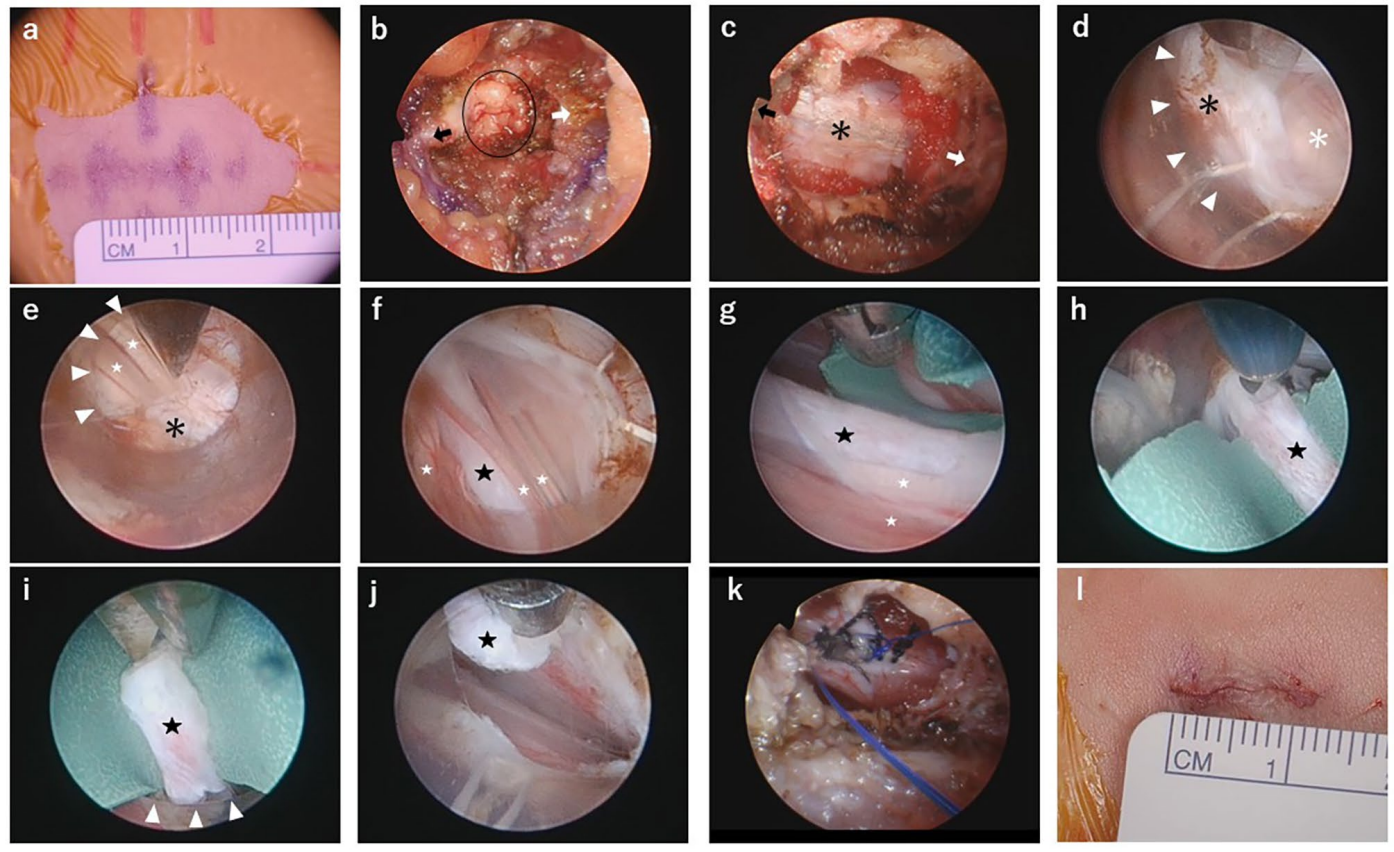
Intraoperative view. **a**: Marking the skin incision. **b**: Resection of interspinous ligament. The epidural fat (black circle) is visible between the L4 lamina (black arrow) and L5 lamina (white arrow). **c**: Exposure of epidural space. The black asterisk is dura. **d**: Dural incision reveals the arachnoid (white asterisk). White triangular arrows indicate the sheath edge. **e**: Limiting deviation of the normal cauda equina nerve roots (white stars) using sheath edge and irrigation. **f**: The black star is the filum terminale. **g**: Green rubber sheet inserted under the filum. **h**: Coagulating of the filum. **i**: Cutting. **j**: Part of the filum removed. **k**: Dural suturing. **l**: Closed incision

The patient underwent surgery in the prone position. Neurophysiological intraoperative electromyographic (EMG) monitoring and monitoring of the bulbocavernosus reflex (BCR) were used. A midline 1 cm skin incision was made at the L4/5 vertebral level (Fig. 2a). The procedures were performed using two types of endoscopes (2.7 mm diameter, 30 degrees, Olympus, Japan; Oi HandyPro, Storz, Germany). A subperiosteal dissection of the muscles on the caudal side of L4 lamina and cranial side of L5 lamina was done, and the dissection was extended to the adjacent interspinous ligament to expose the ligamentum flavum (Fig. 2b).

Next, the interspinous ligament and partial lamina were removed and the epidural fat was exposed. The epidural fat was dissected and moved into the space under the surrounding bone and ligaments (Fig. 2c). We used a 6 mm diameter Smart Needle Sheath (Fujisystems, Japan) as a tubular retractor. After sheath cannulation, a midline durotomy of 6 mm was performed with a bipolar coagulation electrode and scissors (Fig. 2d).

The key to incising the arachnoid membrane was limiting the deviation of the normal cauda equina nerve roots using the sheath edges and irrigation (Fig. 2e). The filum terminale was identified by its thickness and pale appearance. (Fig. 2f). We placed a small green rubber sheet as an underlay to anchor the filum and protect the normal caudal nerve roots. (Fig. 2g). We stimulated the filum to confirm the absence of a motor response. The filum was resected cranial side first, carefully maintaining hemostasis (Fig. 2 h, 2i). After releasing the filum, it retracted rapidly cranially. The caudal side of the filum was cut, and part of the filum was removed and sent for pathological examination. (Fig. 2j). After removing the sheath, the dura and arachnoid membrane were sutured together with 4 − 0 nylon. (Fig. 2k). The wound was closed in layers. (Fig. 2l).

For extradural procedures, excellent image quality and a spacious working environment are indispensable, especially for tasks involving the excision of tough tissues like bone and ligaments, as well as dural suturing. Therefore, we employed a 30-degree Olympus endoscope (Fig. 2b, c and k).

On the other hand, intradural procedures, though relatively straightforward in terms of manual techniques, necessitate precise control of cerebrospinal fluid. To address this need, we utilized a Storz endoscope, which enabled us to perform the procedure while simultaneously infusing artificial cerebrospinal fluid (Fig. 2d g).

### Postoperative course

**Fig. 3:**
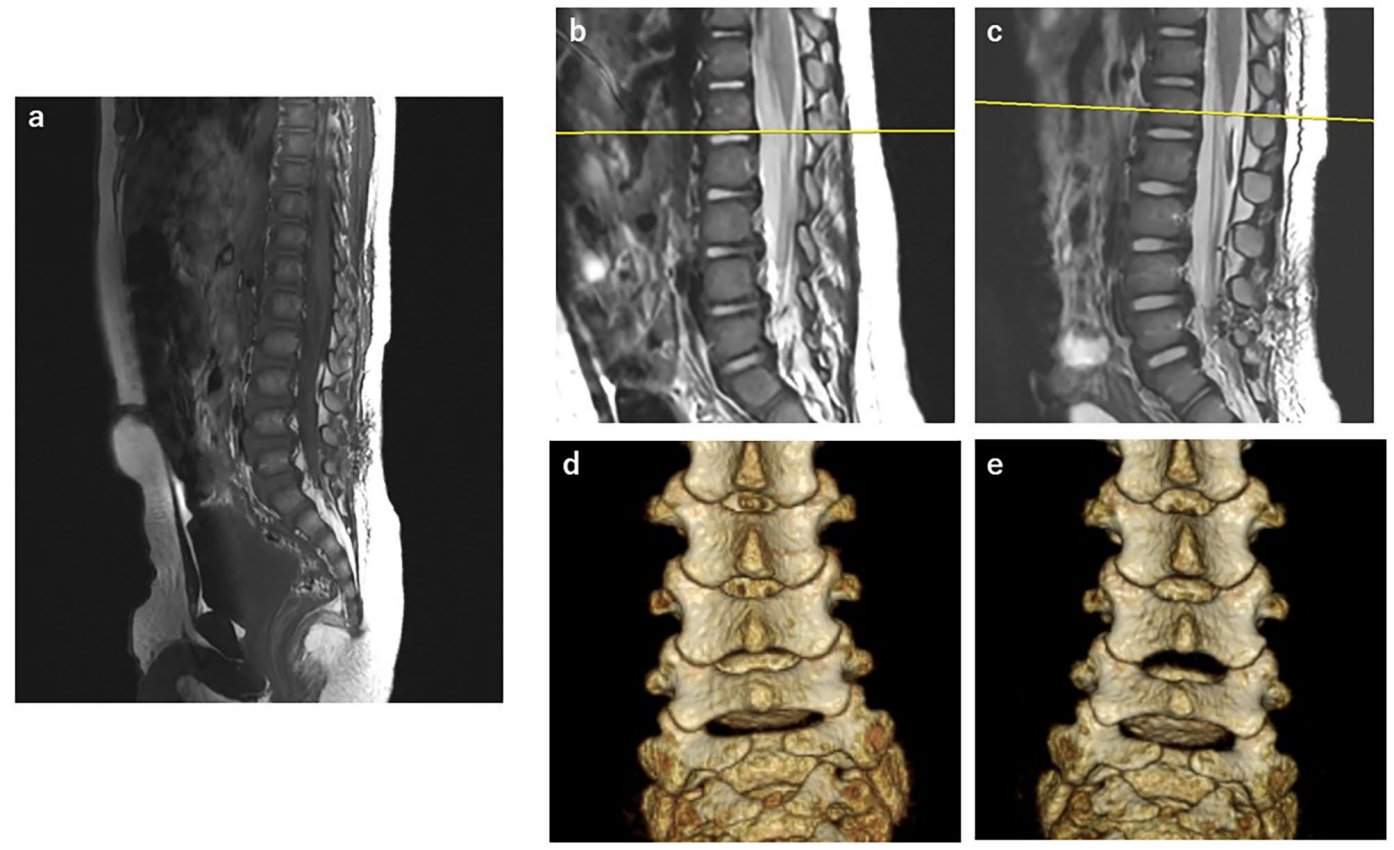
Postoperative Findings. **a**: Postoperative MRI showed cranial shift of the resected filum terminale. **b**: Preoperative sagittal T2-weighted image showed the conus level at lower L2. **c**: Postoperative sagittal T2-weighted image showed the conus level at middle L2. **d**: Preoperative 3-dimensional computed tomography (3DCT) image of the lumbar spine. **e**: Postoperative 3DCT image of the lumbar spine

The postoperative course was uneventful. Postoperative MRI revealed that the filum terminale had resected and the spinal conus level had shifted cranially. (Fig. 3a, b and c). A comparison of pre- and postoperative 3-dimensional computed tomography images demonstrated that the L4,5 vertebral arches were less affected (Fig. 3d and e). The patient remained neurologically intact and was discharged 4 days after surgery.

## Discussion and conclusions

Untethering by sectioning the filum terminale for tethered cord syndrome is almost always performed on children [1–4]. Furthermore, prophylactic surgery is sometimes performed for asymptomatic patients. Therefore, the surgical invasiveness should be as limited as possible, and surgical techniques that minimize complications without compromising safety or efficacy are necessary.

In microscopic surgery, the surgeon’s hands and instruments can obstruct the view, so the incision area tends to be wide to ensure visibility. However, endoscopic surgery requires only the working space of the endoscope and tools, allowing for a small skin incision and surgical corridor.

Endoscopic surgery also increases certainty and safety because it allows close observation of the operative site. For example, when it is difficult to detect the filum terminale due to a small surgical corridor or durotomy, inserting an endoscope into the dura mater can detect the filum terminale reliably without expanding the surgical area. The use of an oblique-viewing endoscope further simplifies the procedure. An oblique-viewing endoscope is also useful for checking for bleeding or adhesions at the end of the cranially retracted filum terminale. Okay O et al. reported that endoscopic release of the filum terminale may provide benefits such as shorter hospital stay and less blood loss [12].

The classical translaminar approach with laminectomy or laminotomy is an anatomically clear and reliable method. It is also versatile and can be used with other spina bifida occulta. The interlaminar approach has some difficulties in surgical technique and understanding of anatomical structures, but it can result in smaller incisions, less invasive surgery, less postoperative pain, and faster recovery [5, 9].

Both endoscopic and interlaminar approaches have reported benefits to patients due to their minimally invasive nature, and it can be inferred that our method of combining both is a better approach. In terms of visibility and minimizing invasiveness, our surgical technique of using the interlaminar approach with endoscopy allows for untethering by sectioning the filum terminale through a very small skin incision. To our knowledge, a 1 cm skin incision for this procedure has not been previously reported. In addition, this report is considered highly unique in two aspects: the modification and combination of endoscopy and the interlaminar approach, particularly in the endoscopic techniques used for intradural operations.　Delayed complications such as re-tethering have not been studied and require long-term follow-up in a greater number of cases.

Our report emphasizes a 1 cm option for minimally invasive untethering by sectioning the filum terminale. In conclusion, there is an urgent need for endoscopes and technology that will lead to further evolution of surgical techniques and benefits to patients.

## Data Availability

Data sharing is not applicable to this article as no datasets were generated or analysed during the current study.

## List of abbreviations

EMG: Electromyography
MRI: Magnetic resonance imaging
